# 1055. ARGONAUT-IV: Susceptibility of Carbapenem-resistant *Klebsiellae* to Ceftibuten/VNRX-5236

**DOI:** 10.1093/ofid/ofab466.1249

**Published:** 2021-12-04

**Authors:** Andrew R Mack, Christopher Bethel, Steven Marshall, Robin Patel, Robin Patel, David van Duin, Vance G Fowler, Daniel D Rhoads, Michael Jacobs, Focco van den Akker, David A Six, Greg Moeck, Krisztina M Papp-Wallace, Robert A Bonomo

**Affiliations:** 1 Case Western Reserve University & Louis Stokes Cleveland VA Medical Center, Cleveland, Ohio; 2 Louis Sokes Cleveland VA Medical Center, Cleveland, OH; 3 Louis Stokes Cleveland Medical Center, Cleveland, OH; 4 Mayo Clinic, Rochester, MN; 5 University of North Carolina, Chapel Hill, North Carolina; 6 Duke University, Durham, North Carolina; 7 Cleveland Clinic, Cleveland, Ohio; 8 University Hospital Cleveland Medical Center, Cleveland, OH; 9 Case Western Reserve University, Cleveland, Ohio; 10 Venatorx Pharmaceuticals, Inc., Malvern, Pennsylvania; 11 Venatorx Pharmaceuticals, Malvern, Pennsylvania; 12 Louis Stokes Cleveland VAMC and Case Western Reserve University, Cleveland, OH; 13 Louis Stokes Cleveland VA Medical Center, Cleveland, OH

## Abstract

**Background:**

Carbapenem resistance in *Klebsiellae* spp. arises through mutational and acquired mechanisms and is considered an “urgent threat” by the CDC. VNRX-5236 is a bicyclic boronate β-lactamase inhibitor (BLI) that combines oral bioavailability (via etzadroxil prodrug VNRX-7145; Figure 1) and activity against all three Ambler classes of serine β-lactamases. VNRX-7145 is currently in development with the oral cephalosporin, ceftibuten (CTB) (Figure 1).

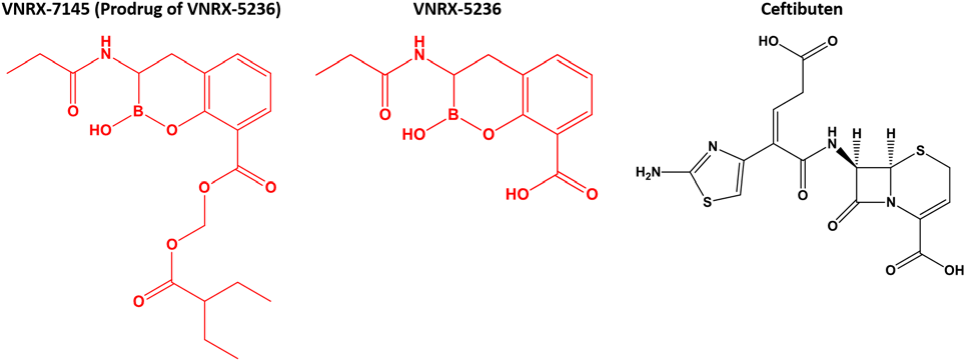

Figure 1. Structures of VNRX-7145, VNRX-5236, and ceftibuten. The β-lactamase inhibitors are in red and the β-lactam antibiotic is in black.

**Methods:**

The activity of CTB/VNRX-5236 against 200 carbapenem-resistant *Klebsiellae* from the Consortium on Resistance against Carbapenems in *Klebsiella* (CRACKLE) was assessed in this study. Among these, 193 expressed class A KPC enzymes, one expressed a class B NDM enzyme, and six expressed a class D OXA-48 or variant enzyme. Minimum inhibitory concentrations (MIC) were determined by broth microdilution (CLSI M07 Ed. 11) using the ThermoFisher Sensititre system with custom assay panels. MICs were interpreted using CLSI M100 Ed. 30, except the EUCAST breakpoint for CTB (S≤1 µg/mL) was used for CTB and was applied for comparative purposes to CTB/VNRX-5236 MICs where VNRX-5236 was fixed at 4 µg/mL. American Type Culture Collection strains were used for quality control.

**Results:**

92.5% of stains studied in this CRACKLE collection were provisionally susceptible to CTB/VNRX-5236. In comparison, strains were 95.5% and 98% susceptible to meropenem-vaborbactam (MVB) and ceftazidime-avibactam (CZA), respectively. MIC_50_s were in the susceptible range for CZA, MVB, and CTB/VNRX-5236; and resistant for CTB, ceftazidime (CAZ) and meropenem (MEM). MIC_90_s were in the susceptible range for CZA, MVB, and CTB/VNRX-5236 and resistant range for CAZ, MEM, and CTB (Table 1). One of four CZA-resistant and three of nine MVB non-susceptible strains were provisionally susceptible to CTB/VNRX-5236.

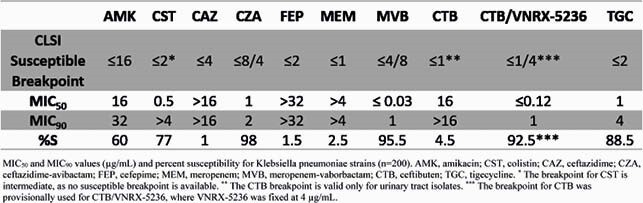

MIC50 and MIC90 values (µg/mL) and percent susceptibility for Klebsiella pneumoniae strains (n=200). AMK, amikacin; CST, colistin; CAZ, ceftazidime; CZA, ceftazidime-avibactam; FEP, cefepime; MEM, meropenem; MVB, meropenem-vaborbactam; CTB, ceftibuten; TGC, tigecycline. * The breakpoint for CST is intermediate, as no susceptible breakpoint is available. ** The CTB breakpoint is valid only for urinary tract isolates. *** The breakpoint for CTB was provisionally used for CTB/VNRX-5236, where VNRX-5236 was fixed at 4 µg/mL.

**Conclusion:**

The addition of VNRX-5236 enhanced the activity of CTB against the 200 *Klebsiella* isolates tested, reaching a total of 92.5% susceptibility. The prodrug (VNRX-7145) allows for oral administration, making it a potential option for step-down therapy. Importantly, VNRX-5236 has a broader spectrum of activity than existing oral BLIs, opening new treatment options for resistant infections as a key addition to the existing antibiotic arsenal.

**Disclosures:**

**Robin Patel, MD**, **1928 Diagnostics** (Consultant)**BioFire Diagnostics** (Grant/Research Support)**ContraFect Corporation** (Grant/Research Support)**Curetis** (Consultant)**Hylomorph AG** (Grant/Research Support)**IDSA** (Other Financial or Material Support, Editor’s Stipend)**Infectious Diseases Board Review Course** (Other Financial or Material Support, Honoraria)**Mammoth Biosciences** (Consultant)**NBME** (Other Financial or Material Support, Honoraria)**Netflix** (Consultant)**Next Gen Diagnostics** (Consultant)**PathoQuest** (Consultant)**PhAST** (Consultant)**Qvella** (Consultant)**Samsung** (Other Financial or Material Support, Patent Royalties)**Selux Diagnostics** (Consultant)**Shionogi & Co., Ltd.** (Grant/Research Support)**Specific Technologies** (Consultant)**TenNor Therapeutics Limited** (Grant/Research Support)**Torus Biosystems** (Consultant)**Up-to-Date** (Other Financial or Material Support, Honoraria) **Robin Patel, MD**, BioFire (Individual(s) Involved: Self): Grant/Research Support; Contrafect (Individual(s) Involved: Self): Grant/Research Support; IDSA (Individual(s) Involved: Self): Editor’s stipend; NBME, Up-to-Date and the Infectious Diseases Board Review Course (Individual(s) Involved: Self): Honoraria; Netflix (Individual(s) Involved: Self): Consultant; TenNor Therapeutics Limited (Individual(s) Involved: Self): Grant/Research Support; to Curetis, Specific Technologies, Next Gen Diagnostics, PathoQuest, Selux Diagnostics, 1928 Diagnostics, PhAST, Torus Biosystems, Mammoth Biosciences and Qvella (Individual(s) Involved: Self): Consultant **David van Duin, MD, PhD**, **Entasis** (Advisor or Review Panel member)**genentech** (Advisor or Review Panel member)**Karius** (Advisor or Review Panel member)**Merck** (Grant/Research Support, Advisor or Review Panel member)**Pfizer** (Consultant, Advisor or Review Panel member)**Qpex** (Advisor or Review Panel member)**Shionogi** (Grant/Research Support, Scientific Research Study Investigator, Advisor or Review Panel member)**Utility** (Advisor or Review Panel member) **Vance G. Fowler, Jr., MD, MHS**, **Achaogen** (Consultant)**Advanced Liquid Logics** (Grant/Research Support)**Affinergy** (Consultant, Grant/Research Support)**Affinium** (Consultant)**Akagera** (Consultant)**Allergan** (Grant/Research Support)**Amphliphi Biosciences** (Consultant)**Aridis** (Consultant)**Armata** (Consultant)**Basilea** (Consultant, Grant/Research Support)**Bayer** (Consultant)**C3J** (Consultant)**Cerexa** (Consultant, Other Financial or Material Support, Educational fees)**Contrafect** (Consultant, Grant/Research Support)**Debiopharm** (Consultant, Other Financial or Material Support, Educational fees)**Destiny** (Consultant)**Durata** (Consultant, Other Financial or Material Support, educational fees)**Genentech** (Consultant, Grant/Research Support)**Green Cross** (Other Financial or Material Support, Educational fees)**Integrated Biotherapeutics** (Consultant)**Janssen** (Consultant, Grant/Research Support)**Karius** (Grant/Research Support)**Locus** (Grant/Research Support)**Medical Biosurfaces** (Grant/Research Support)**Medicines Co.** (Consultant)**MedImmune** (Consultant, Grant/Research Support)**Merck** (Grant/Research Support)**NIH** (Grant/Research Support)**Novadigm** (Consultant)**Novartis** (Consultant, Grant/Research Support)**Pfizer** (Grant/Research Support)**Regeneron** (Consultant, Grant/Research Support)**sepsis diagnostics** (Other Financial or Material Support, Pending patent for host gene expression signature diagnostic for sepsis.)**Tetraphase** (Consultant)**Theravance** (Consultant, Grant/Research Support, Other Financial or Material Support, Educational fees)**Trius** (Consultant)**UpToDate** (Other Financial or Material Support, Royalties)**Valanbio** (Consultant, Other Financial or Material Support, Stock options)**xBiotech** (Consultant) **Daniel D. Rhoads, MD**, **Becton, Dickinson and Company** (Grant/Research Support) **Michael Jacobs, MBBS**, **Venatorx Pharmaceuticals, Inc.** (Grant/Research Support) **Focco van den Akker, PhD**, **Venatorx Pharmaceuticals, Inc.** (Grant/Research Support) **David A. Six, PhD**, **Venatorx Pharmaceuticals, Inc.** (Employee) **Greg Moeck, PhD**, **Venatorx Pharmaceuticals, Inc.** (Employee) **Krisztina M. Papp-Wallace, Ph.D.**, **Merck & Co., Inc.** (Grant/Research Support)**Spero Therapeutics, Inc.** (Grant/Research Support)**Venatorx Pharmaceuticals, Inc.** (Grant/Research Support)**Wockhardt Ltd.** (Other Financial or Material Support, Research Collaborator) **Robert A. Bonomo, MD**, **entasis** (Research Grant or Support)**Merck** (Grant/Research Support)**NIH** (Grant/Research Support)**VA Merit Award** (Grant/Research Support)**VenatoRx** (Grant/Research Support)

